# Lack of clinically important PK interaction between coformulated ledipasvir/sofosbuvir and rilpivirine/emtricitabine/tenofovir alafenamide

**DOI:** 10.1002/prp2.353

**Published:** 2017-09-11

**Authors:** Joseph M. Custodio, Susan K. Chuck, Hoa Chu, Huyen Cao, Grace Ma, John Flaherty, John Ling, Brian P. Kearney

**Affiliations:** ^1^ Gilead Sciences 333 Lakeside Drive Foster City California 94404

**Keywords:** Coinfection, drug interaction, HCV, HIV, ledipasvir, pharmacokinetics, rilpivirine, sofosbuvir, tenofovir alafenamide

## Abstract

The drug–drug interaction (DDI) potential between the fixed‐dose combinations of ledipasvir/sofosbuvir 90/400 mg for hepatitis C virus and emtricitabine/rilpivirine/tenofovir alafenamide (TAF) 200/25/25 mg for HIV was evaluated in a randomized, open‐label, single‐center, multiple‐dose, 3‐way, 6‐sequence, crossover Phase 1 study in 42 healthy subjects. Emtricitabine/rilpivirine/TAF had no relevant effect on the pharmacokinetic parameters of maximum concentration [*C*_max_] and area under the concentration versus time curve over the dosing interval [AUC_tau_] for ledipasvir, sofosbuvir, and the metabolites GS‐566500 and GS‐331007. Ledipasvir/sofosbuvir had no effect on the *C*_max_ and AUC_tau_ for rilpivirine and emtricitabine. The *C*_max_ and AUC_tau_ of tenofovir, the major metabolite of TAF, were increased by 62% and 75%, respectively. However, the resulting absolute tenofovir exposures were markedly lower than the historical tenofovir exposures following tenofovir disoproxil fumarate (TDF) and, as such, were not considered to be clinically relevant. In contrast, additional adverse effect monitoring is recommended upon coadministration of ledipasvir and TDF due to elevated tenofovir exposures resulting from the DDI. This difference is explained by the fact that TAF 25 mg results in markedly lower (~90%) plasma tenofovir exposure compared to TDF 300 mg. Ledipasvir/sofosbuvir and emtricitabine/rilpivirine/TAF were generally well tolerated when administered alone or in combination. HIV/hepatitis C virus‐coinfected patients can coadminister ledipasvir/sofosbuvir and emtricitabine/rilpivirine/TAF without dosage adjustments.

**Abbreviations**
AE: adverse event
ART: antiretroviral therapy
ARV: antiretroviral
AUC: area under the curve
BCRP: breast cancer resistance protein
BLQ: below the limit of quantitation
BMI: body mass index
CrCl: creatinine clearance
CYP: cytochrome P450
DAA: direct‐acting antivirals
DDI: drug–drug interaction
FDC: fixed‐dose combination
GLSM: geometric least‐squares mean
HCV: hepatitis C virus
HIV: human immunodeficiency virus
LDV: ledipasvir
PK: pharmacokinetic
RPV: rilpivirine
SOF: sofosbuvir
TAF: tenofovir alafenamide
TDF: tenofovir disoproxil fumarate
TFV: tenofovir

## Introduction

In the United States, coinfection with human immunodeficiency virus (HIV) and hepatitis C virus (HCV) is common, as approximately 25% of HIV‐infected persons are harboring HCV (Centers for Disease Control and Prevention, 2017). The prevalence of HIV/HCV coinfection is more common (50–90%) in people who are both HIV‐infected and inject drugs. Coinfected individuals are more likely to have liver‐related morbidity and mortality, non‐hepatic organ dysfunction, and a higher overall mortality than individuals who are monoinfected with HCV (Chen et al. 2009; Lo Re et al. 2014). Despite advances in antiretroviral (ARV) regimens, infection with HIV continues to be independently associated with advanced liver fibrosis and cirrhosis in HIV/HCV‐coinfected patients (de Ledinghen et al. 2008; Thein et al. 2008; Fierer et al. 2013; Kirk et al. 2013). Direct‐acting antivirals (DAAs) have reshaped the treatment of HCV by their improved efficacy and safety, significantly shortened treatment duration, and elimination of the need for pegylated interferon and ribavirin in most instances. With more coinfected patients seeking treatment for chronic HCV, drug–drug interactions (DDIs) between DAAs and antiretroviral therapies (ARTs) are important to explore because of their potential to impact HIV and HCV treatment decisions.

The coadministration of tenofovir disoproxil fumarate (TDF) with ledipasvir (LDV) has resulted in elevated tenofovir (TFV) exposure documented in pharmacokinetic (PK) studies and clinical trials (German et al. 2014; Mathias 2015; Bunnell et al. 2016). The highest absolute TFV exposures were observed when the fixed‐dose combination (FDC) LDV/sofosbuvir (SOF) and ritonavir‐boosted HIV protease inhibitors with emtricitabine (FTC)/TDF were coadministered (German et al. 2015) with the resultant TFV exposures beyond the known exposure–safety range of TFV. Given these PK findings and concerns, increased monitoring for TDF adverse reactions or use of alternative HCV or HIV therapy is recommended (Gilead Sciences Inc, 2017a). Tenofovir alafenamide (TAF), a prodrug of TFV associated with 80–90% less plasma TFV exposures than TDF due to the lower dosage, is an alternative to TDF for HIV management in HIV/HCV‐coinfected individuals (Sax et al. 2015; Zack et al. 2016; Gilead Sciences Inc, 2017b). The majority of TAF is delivered to peripheral blood mononuclear cells intact due to the greater stability of TAF in plasma compared to TDF. The introduction of TAF‐based FDCs, which provide an equally effective yet safer option than TDF‐based FDCs for HIV patients, warrants the evaluation of potential DDIs of these regimens with LDV and SOF.

LDV/SOF is indicated with or without ribavirin for the treatment of chronic HCV genotype 1, 4, 5, or 6 (Gilead Sciences Inc, 2017c). LDV is a HCV nonstructural protein (NS)5A inhibitor, which is eliminated mainly unchanged via biliary excretion (Gilead Sciences Inc, 2017c). SOF is a HCV nucleotide analog NS5B polymerase inhibitor; its metabolites are both inactive, GS‐566500 (intermediate metabolite) and GS‐331007 (predominant circulating metabolite) (Denning et al. 2013). LDV inhibits the drug transporters P‐glycoprotein (P‐gp) and breast cancer resistance protein (BCRP), and may increase the plasma concentrations of substrates for these transporters (German et al. 2012). LDV and SOF are not inducers or inhibitors of cytochrome P450 (CYP) and are unlikely to be involved in CYP‐mediated interactions. Both LDV and SOF (not GS‐331007) are substrates of P‐gp and BCRP, and their plasma concentrations may be reduced by inducers potentially leading to HCV treatment failure or increased by inhibitors of these transporters (Kirby et al. 2012; Mathias et al. 2012; German et al. 2012).

Rilpivirine (RPV), FTC, and TAF are available as a FDC R/F/TAF for the treatment of HIV‐1 (Gilead Sciences Inc, 2017d). RPV is a non‐nucleoside reverse transcriptase inhibitor and is primarily metabolized by CYP3A. The approved 25 mg dose of RPV does not affect CYP or P‐gp. FTC and TAF are both nucleoside analog reverse transcriptase inhibitors. FTC is not significantly metabolized and is eliminated by glomerular filtration and active tubular secretion. TAF is a substrate of P‐gp, BCRP, OATP1B1, and OATP1B3; TAF is a weak inhibitor of CYP3A in vitro. TAF is metabolized to TFV by cathepsin A in peripheral blood mononuclear cells and carboxylesterase 1 in hepatocytes. TAF is minimally metabolized by CYP3A.

HIV and HCV infections both require multidrug regimens for effective treatment, which increases the risk of DDI when managing HIV/HCV‐coinfected patients. One potential concern with coadministration of LDV/SOF with TDF‐ and TAF‐containing regimens is the potential for the inhibition of P‐gp by LDV (P‐gp inhibitor) as TDF and TAF are P‐gp substrates. Although FDCs have been shown to improve adherence in HIV or HCV treatment, they are limited by the inability to adjust doses of the individual components (Gardner et al. 2005; Portsmouth et al. 2005; Petersen et al. 2006; Sax et al. 2011, 2012). The PK study presented here was conducted to evaluate the potential for DDIs between LDV/SOF 90/400 mg and R/F/TAF 25/200/25 mg to expand treatment options for HIV/HCV‐coinfected patients.

## Materials and Methods

### Study population

HIV‐ and HCV‐negative male and non‐pregnant, non‐lactating female subjects between 18 and 45 years of age (inclusive), with a body mass index (BMI) between 19.0 and 30.0 kg/m^2^ (inclusive), and in general good health were enrolled into the study. Subjects were required to have a creatinine clearance (CrCl) ≥70 mL/min (using Cockcroft–Gault formula and actual body weight).

All screening laboratory evaluations (hematology, chemistry, and urinalysis) had to be within normal range, and subjects who had syncope, palpitations, or unexplained dizziness; who had an implanted defibrillator or pacemaker; or who had any serious or active medical or psychiatric illness were excluded. They were also excluded if they took any prescription medications or over‐the‐counter medications including herbal products within 28 days of commencing study drug dosing; exceptions were vitamins, acetaminophen, ibuprofen, and/or hormonal contraceptive medications. Additionally, subjects treated with systemic steroids, immunosuppressant therapies, or chemotherapeutic agents within 3 months of study screening were excluded. Subjects with current alcohol or substance abuse, judged by the investigator to potentially interfere with compliance or compromise safety, were excluded. Subjects were restricted, both before the first dose of study drug and through to discharge, from consuming alcohol‐containing products; using nicotine‐containing products; and consuming grapefruit juice, grapefruits, and Seville orange juice. Consuming caffeine and other methyl xanthines were prohibited on dosing days.

Informed consent was obtained from each subject before initiation of study procedures. The study protocols and consent forms were reviewed and approved by a duly constituted institutional review board (Schulman IRB, Research Triangle Park, NC). The study was performed in accordance with the principles of Good Clinical Practice and the Declaration of Helsinki and was consistent with the requirements of the US Code of Federal Regulations Title 21, Part 312.

### Study design

This study was a randomized, open‐label, single‐center, multiple‐dose, 3‐way, 6‐sequence, crossover Phase 1 study in healthy adults under fed conditions (approximately 600 calories, 27% fat). The treatments were LDV/SOF 90/400 mg (Treatment A), R/F/TAF 25/200/25 mg (Treatment B), and LDV/SOF+R/F/TAF (Treatment C). Subjects were randomized to 1 of 6 treatment sequences as described in Table 1.

**Table 1:** Summary of treatment sequences

Sequence	Days 1–11	Days 12–22	Days 23–33
1	LDV/SOF	R/F/TAF	LDV/SOF + R/F/TAF
2	LDV/SOF	LDV/SOF + R/F/TAF	R/F/TAF
3	R/F/TAF	LDV/SOF + R/F/TAF	LDV/SOF
4	R/F/TAF	LDV/SOF	LDV/SOF + R/F/TAF
5	LDV/SOF + R/F/TAF	R/F/TAF	LDV/SOF
6	LDV/SOF + R/F/TAF	LDV/SOF	R/F/TAF

LDV/SOF, ledipasvir/sofosbuvir; R/F/TAF, rilpivirine/emtricitabine/tenofovir alafenamide.

Patients were administered the study drug within 5 minutes of completing the standardized breakfast. Serial blood samples for PK assessments were collected on the last day of each dosing sequence at the following time points: predose (≤5 min), 0.25, 0.5, 0.75, 1, 1.5, 2, 3, 4, 4.5, 5, 6, 8, 10, 12, 18, and 24 hours after the administration of the dose. Subjects were restricted from food intake until after the 4‐hour PK blood sampling time point.

### Bioanalytic methods

Bioanalysis was conducted at QPS, LLC (Newark, DE). Plasma concentrations of LDV, SOF, GS‐566500, GS‐331007, RPV, FTC, TAF, and TFV were determined by validated high‐performance liquid chromatography/tandem mass spectrometry (HPLC–MS/MS) methods with multiple reaction monitoring and electrospray ionization in the positive mode for LDV, RPV, FTC, TAF, and TFV, and in the negative mode for SOF, GS‐566500, and GS‐331007. Isotopically labeled internal standards (^2^H, ^13^C, and/or ^15^N) were used for each analyte. Response ratios of analyte:internal standard observed for calibration standards were subjected to linear regression with 1/(nominal concentration)^2^ weighting, and the concentration of an analyte in a sample was determined by interpolation of its response ratio in the standard curve equation. Concentrations below the calibrated ranges of the methods were reported as below the limit of quantitation (BLQ); samples with concentrations above the calibrated ranges of the methods were diluted with blank plasma prior to analysis and the appropriate dilution factor was applied to the result. For each method, the results of within‐run (intra‐assay) and between‐run (inter‐assay) precision assessments were reported as the coefficients of variation, each expressed as a percentage (%CV), and the results of accuracy assessments were reported as the relative error values expressed as percentages (%RE). Interference testing demonstrated that no analyte interfered in the quantification of any other analyte by any of the methods used for the study. The calibrated ranges of the method were 1–2000 ng/mL for LDV, 5–2500 ng/mL for SOF, 10–5000 ng/mL for GS‐566500 and GS‐331007, 1 to 500 ng/mL for RPV, 5 to 3000 ng/mL for FTC, 1 to 1000 ng/mL for TAF, and 0.3–300 ng/mL for TFV. For all eight analytes, all %CV values were <9.8% and all %RE values were within ±6.9% of 100%.

### Safety assessments

Safety was evaluated throughout the study and assessments included reviews of adverse events (AEs) and concomitant medications, clinical laboratory analyses, vital sign measurements, and physical examinations. Treatment‐emergent AEs were defined as any event with an onset date of on or after the study drug start date and up to 30 days after the permanent discontinuation of study drug. Clinical and laboratory AEs were coded using the Medical Dictionary for Regulatory Activities (MedDRA) version 18. The severity of AEs was graded according to the Gilead Sciences, Inc. Grading Scale for Severity of Adverse Events and Laboratory Abnormalities (grades 1–4).

### Pharmacokinetic analysis

The PK analysis sets included all randomized subjects who received at least 1 dose of study drug and had at least 1 plasma concentration data point for each analyte. Samples BLQ of bioanalytical assays occurring before the achievement of the first quantifiable concentration were assigned a value of zero to prevent overestimation of the initial area under the plasma concentration–time curve (AUC) and at all other time points were treated as missing data in WinNonlin. For summary statistics, samples BLQ at predose time were zero and postdose time points were assigned one‐half the value of the lower limit of quantitation.

Pharmacokinetic parameters were estimated using standard non‐compartmental methods in conjunction with the linear/log trapezoidal rule (Phoenix WinNonlin^®^, version 6.3; Certara USA, Inc., Princeton, NJ). The primary PK parameters were area under the plasma concentration versus time curve over the dosing interval (AUC_tau_), area under the plasma concentration versus time curve from zero to the last quantifiable concentration (AUC_last_), maximum observed plasma concentration (*C*_max_), and observed drug concentration at the end of the dosing interval (*C*_tau_) depending on the analytes. The following PK parameters were also calculated: time of maximum observed plasma concentration (*T*_max_) and elimination half‐life of the drug in plasma (*t*_1/2_).

### Statistical analysis

The primary endpoints were the PK parameters AUC_tau_, *C*_max_, and *C*_tau_ of RPV, FTC, LDV, and GS‐331007; AUC_last_ and *C*_max_ of TAF; AUC_tau_ and *C*_max_ of SOF and GS‐566500. The secondary endpoints were the PK parameters AUC_tau_, *C*_max_, and *C*_tau_ of TFV; *T*_max_, C_last_, and T_last_ of RPV, FTC, TAF, TFV, LDV, SOF, GS‐566500, and GS‐331007. No clinically significant interaction between LDV/SOF and R/F/TAF was concluded if the 90% confidence intervals (CIs) for the geometric least‐squares mean (GLSM) ratios for the primary PK parameters of RPV were within 80% and 125% and of other analytes were within the boundaries of 70% and 143%.

At least 36 evaluable subjects or 6 evaluable subjects per sequence were needed to achieve 90% CIs for the GLSM ratios of the test versus reference treatments within 80% and 125%, with regards to the primary PK parameters for RPV (85% chance if the estimated GLSM ratio was 100%), and within 70–143%, with regard to the primary PK parameters for FTC, TAF, TFV, SOF, GS‐566500, GS‐331007, and LDV (90% chance if the estimated GLSM ratio was 100%). SAS software (SAS Institute, Cary, North Carolina, USA) was used to perform the statistical summaries and analyses.

Subject demographic data and baseline characteristics were summarized by sequence using descriptive statistics. Plasma concentrations and PK parameters were summarized by treatment using descriptive statistics. For each analyte and PK parameter, a parametric mixed‐effects analysis of variance (ANOVA) model was fitted to the natural log‐transformed values of the PK parameter under evaluation using SAS^®^ PROC MIXED; this model included treatment, sequence, and period as fixed effects and subject within sequence as a random effect. SAS^®^ PROC MIXED (SAS Institute, Cary, NC) was used to conduct the treatment comparison analysis and the 90% CI calculations for the natural log‐transformed PK parameters. The safety analysis set included all randomized subjects who received at least 1 dose of study drug, and safety data were collected on the date of the first dose of study drug through 30 days after the last dose of study drug. Safety data including AEs, laboratory data, and vital signs were summarized by treatment, and the incidence of graded AEs and laboratory abnormalities was calculated.

## Results

### Subject demographics and disposition

A total of 42 subjects were randomized and received at least 1 dose of study drug. The majority of subjects were male (71.4%) and white (61.9%). At baseline, the mean age was 34 (range: 18–45), mean BMI was 27.3 kg/m^2^ (range: 22.8–29.9), and mean CrCl by Cockcroft–Gault method was 122.3 mL/min (standard deviation: 19.4). One subject discontinued due an AE of colitis.

### Pharmacokinetics

**Figure 1:**
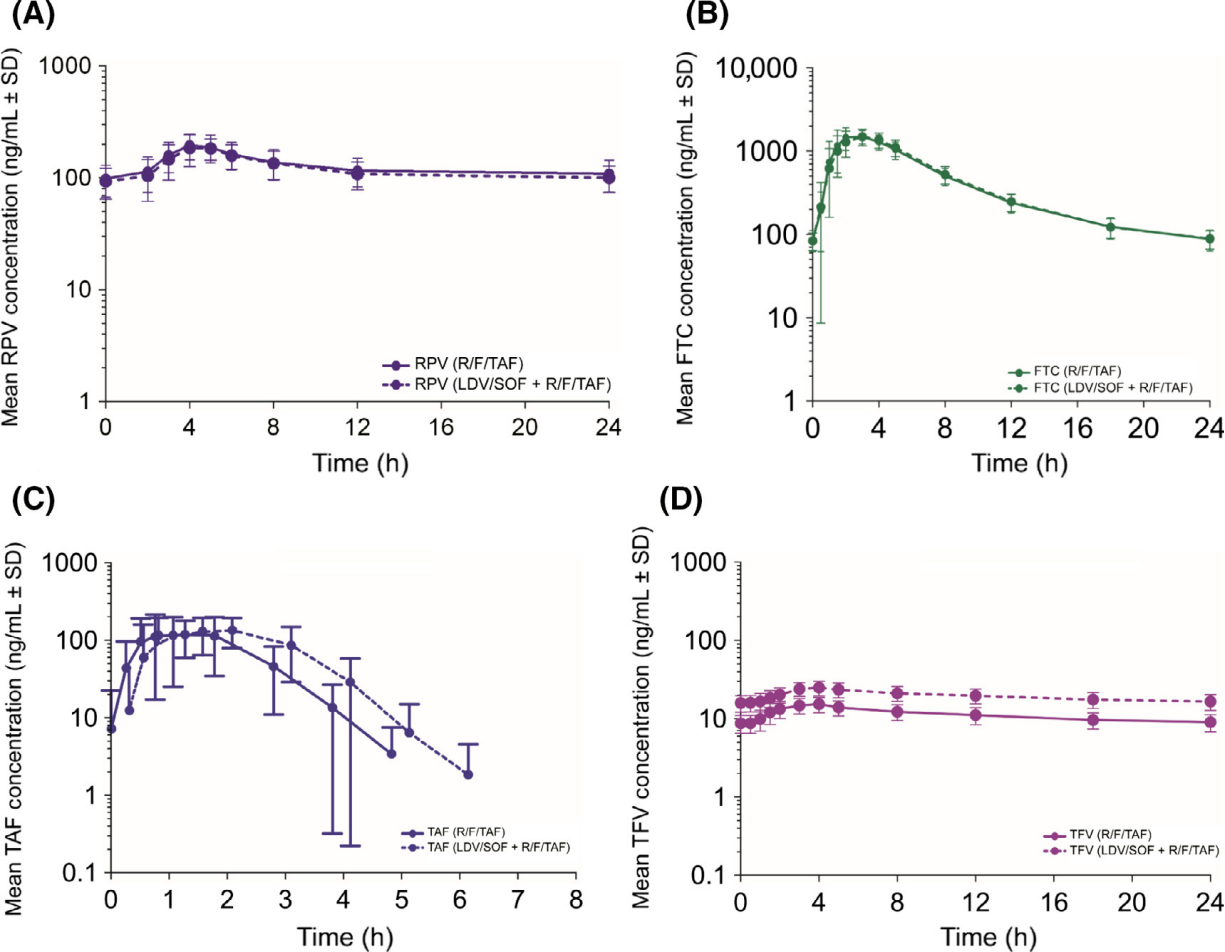
(A–D) Mean (SD) RPV, FTC, TAF, and TFV plasma concentration–time profiles are presented.

**Figure 2:**
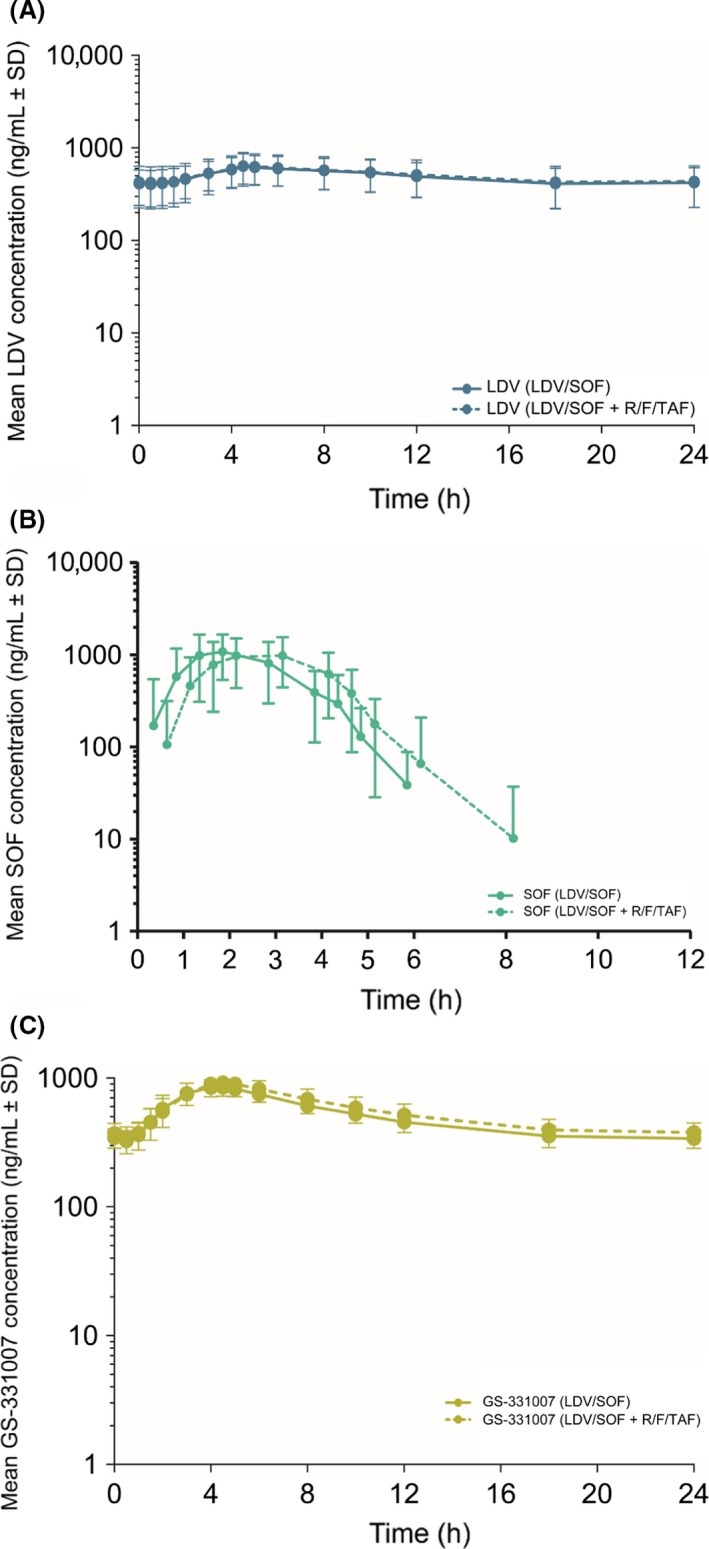
(A–C) Mean (SD) LDV, SOF, and GS‐331007 plasma concentration–time profiles are presented.

The PK analysis sets included 42 subjects. Mean (SD) LDV, SOF, GS‐331007, RPV, FTC, TAF, and TFV plasma concentration–time profiles are presented in Figures 1 and 2. The plasma PK parameters after the administration of the test or reference treatment are presented in Table 2 and include GS‐566500. The statistical analyses of the PK parameters are presented in Table 3.

**Table 2:** Summary of pharmacokinetic parameters

Summary of LDV, SOF, GS‐566500, and GS‐331007 pharmacokinetic parameters								
PK parameter^a^	LDV		SOF		GS‐566500		GS‐331007	
	Test: LDV/SOF + R/F/TAF (*N* = 42)	Reference: LDV/SOF (*N* = 41)	Test: LDV/SOF + R/F/TAF (*N* = 42)	Reference: LDV/SOF (*N* = 41)	Test: LDV/SOF + R/F/TAF (*N* = 42)	Reference: LDV/SOF (*N* = 41)	Test: LDV/SOF + R/F/TAF (*N* = 42)	Reference: LDV/SOF (*N* = 41)
AUC_tau_ (ng·h/mL)	11,945 (43)	11,590 (40)	3069 (31)	2909 (33)	2575 (16)	2504 (16)	12,883 (16)	11,766 (13)
*C*_max_ (ng/mL)	658 (38)	647 (36)	1391 (32)	1470 (35)	502 (18)	510 (20)	960 (15)	884 (14)
*C*_tau_ (ng/mL)	434 (47)	420 (46)	–	–	–	–	378 (19)	340 (16)
*T*_max_ (h)	4.5 (4.5, 5.0)	4.5 (4.5, 5.0)	2.0 (2.0, 3.0)	2.0 (1.5, 3.0)	4.0 (3.0, 4.0)	3.0 (2.0, 4.0)	4.5 (4.0, 5.0)	4.5 (4.0, 4.5)
*t*_1/2_ (h)	31.3 (24.2, 37.5)	32.6 (22.1, 40.6)	0.5 (0.5, 0.6)	0.5 (0.4, 0.6)	2.4 (2.1, 2.9)	2.2 (2.0, 2.5)	16.5 (14.5, 21.8)	16.7 (13.8, 21.9)

Summary of RPV, FTC, TAF, and TFV pharmacokinetic parameters								
PK parameter^a^	RPV		FTC		TAF		TFV	
	Test: LDV/SOF + R/F/TAF (*N* = 42)	Reference: R/F/TAF (*N* = 42)	Test: LDV/SOF + R/F/TAF (*N* = 42)	Reference: R/F/TAF (*N* = 42)	Test: LDV/SOF + R/F/TAF (*N* = 42)	Reference: R/F/TAF (*N* = 42)	Test: LDV/SOF + R/F/TAF (*N* = 42)	Reference: R/F/TAF (*N* = 42)
AUC_tau_ (ng·h/mL)	2858 (26)	3040 (27)	10,805 (15)	10,764 (14)	–	–	467 (21)	268 (23)
AUC_last_ (ng·h/mL)	–	–	–	–	362 (34)	277 (38)	–	–
*C*_max_ (ng/mL)	197 (28)	203 (25)	1650 (18)	1708 (20)	205 (46)	200 (44)	25 (20)	16 (22)
*C*_tau_ (ng/mL)	100 (26)	109 (32)	89 (25)	88 (28)	–	–	17 (22)	9 (25)
*T*_max_ (h)	4.0 (4.0, 5.0)	5.0 (4.0, 5.0)	3.0 (2.0, 3.0)	2.5 (1.5, 3.0)	1.8 (1.0, 2.0)	1.5 (1.0, 2.0)	4.0 (3.0, 4.0)	4.0 (3.0, 4.0)
*t*_1/2_ (h)	30.4 (21.6, 42.5)	32.8 (26.1, 53.0)	5.1 (4.7, 6.9)	6.5 (4.8, 7.7)	0.5 (0.4, 0.5)	0.5 (0.4, 0.6)	44.5 (34.0, 51.9)	32.0 (28.4, 38.0)

%CV, percentage coefficient of variation; AUC_last_, area under the plasma concentration versus time curve from time zero to the last quantifiable concentration; AUC_tau_, area under the plasma concentration versus time curve over the dosing interval; *C*_max_, maximum observed plasma concentration; *C*_tau_, observed drug concentration at the end of the dosing interval; FTC, emtricitabine; LDV, ledipasvir; PK, pharmacokinetic; RPV, rilpivirine; SOF, sofosbuvir; TAF, tenofovir alafenamide; TFV, tenofovir; *T*_max_, time of maximum observed plasma concentration; *t*_1/2_, elimination half‐life of the drug in plasma.

a Data are mean (%CV) except for *T*_max_ and *t*_1/2_, which are reported as median (first quartile, third quartile).

**Table 3:** Statistical comparisons of pharmacokinetic parameters for test versus reference treatments

LDV, SOF, GS‐566500, and GS‐331007 pharmacokinetic parameters for test versus reference treatments								
	LDV		SOF		GS‐566500		GS‐331007	
	GLSM ratio (test/reference) (%)	90% CI (%)	GLSM ratio (test/reference) (%)	90% CI (%)	GLSM ratio (test/reference) (%)	90% CI (%)	GLSM ratio (test/reference) (%)	90% CI (%)
AUC_tau_	102	97, 106	105	101, 109	102	99, 105	108	106, 110
*C*_max_	101	97, 105	96	89, 104	99	95, 103	108	105, 111
*C*_tau_	102	98, 107	–	–	–	–	110	107, 112

RPV, FTC, TAF, and TFV pharmacokinetic parameters for test versus reference treatments								
	RPV		FTC		TAF		TFV	
	GLSM ratio (test/reference) (%)	90% CI (%)	GLSM ratio (test/reference) (%)	90% CI (%)	GLSM ratio (test/reference) (%)	90% CI (%)	GLSM ratio (test/reference) (%)	90% CI (%)
AUC_tau_	95	91, 98	100	98, 102	–	–	175	169, 181
AUC_last_	–	–	–	–	132	125, 140	–	–
*C*_max_	97	92, 102	97	93, 102	103	94, 114	162	156, 168
*C*_tau_	93	89, 97	102	98, 105	–	–	185	178, 192

AUC_last_, area under the plasma concentration versus time curve from time zero to the last quantifiable concentration; AUC_tau_, area under the plasma concentration versus time curve over the dosing interval; CI, confidence interval; *C*_max_, maximum observed plasma concentration; *C*_tau_, observed drug concentration at the end of the dosing interval; FTC, emtricitabine; GLSM, geometric least‐squares mean; LDV, ledipasvir; PK, pharmacokinetic; RPV, rilpivirine; SOF, sofosbuvir; TAF, tenofovir alafenamide; TFV, tenofovir.

The coadministration of LDV/SOF and R/F/TAF did not noticeably increase the exposure of LDV, SOF, or GS‐331007; the 90% CIs for the GLSM ratios for the primary PK parameters were within the protocol predefined boundaries. LDV/SOF had no effect on RPV, FTC, or TAF; the 90% CIs for the GLSM ratios for the primary PK parameters were within the predefined boundaries. However, TFV exposure was increased, with the 90% CIs for the GLSM ratios for AUC_tau_ 175% (169–181%), *C*_max_ 162% (156–168%), and *C*_tau_ 185% (178–192%) outside the predefined boundaries.

### Safety

LDV/SOF and R/F/TAF were generally well tolerated by the study subjects when administered alone or in combination. No Grades 3–4 AEs, serious AEs, or deaths were reported. One subject discontinued due to a Grade 2 nonserious AE of colitis, considered to be study drug related by the investigator. Commonly reported (in at least 2 subjects) treatment‐related AEs were only reported following treatment with LDV/SOF (Treatment A): nausea (5%) and vomiting (5%). The majority of the laboratory abnormalities were Grade 1 or 2 in severity. Three subjects had Grade 3 or 4 laboratory abnormalities, but no corresponding AEs were reported. The Grade 4 laboratory abnormality of increased creatine kinase was transient and consistent with physical exercise.

## Discussion

Overall, LDV/SOF and R/F/TAF coadministration does not require dose adjustment since there is no clinically relevant interaction, despite elevations of TFV PK parameters outside of the protocol predefined lack of interaction boundaries. Notably, all the primary PK parameters of LDV, SOF, GS‐566500, GS‐331007, RPV, FTC, and TAF were within the protocol predefined lack of PK alteration boundaries.

The AASLD and IDSA HCV guidance indicates that LDV/SOF can be used with most ARVs (American Association for the Study of Liver Diseases, 2017); however, due to LDV increasing TFV plasma exposures when given as TDF, concomitant use mandates consideration of CrCl rate and should be avoided in those with CrCl below 60 mL/min. Given the potentiation of this effect when TDF is used with ritonavir or cobicistat‐boosted regimens, LDV should be avoided with this combination (pending further data) unless the ARV regimen cannot be changed and the urgency of treatment is high per the HCV guidance document. The HCV guidance recommends baseline and ongoing assessment for TFV nephrotoxicity for those TDF‐based ARV and DAA combinations which are expected to increase TFV exposures.

LDV/SOF is associated with the fewest ARV drug interactions compared to other DAAs as only tipranavir (rarely used ARV) and TDF (particularly with the PK boosters ritonavir and cobicistat) are concerns and may require ongoing renal monitoring (American Association for the Study of Liver Diseases, 2017; Gilead Sciences Inc, 2017c). In comparison, all other DAAs have more limitations on which ARVs can be coadministered and some require dosage adjustments of the DAAs or HIV regimen (American Association for the Study of Liver Diseases, 2017).

Coadministration of TDF with LDV/SOF has resulted in elevated TFV exposure documented in PK studies and clinical trials (German et al. 2014, 2015; Mathias 2015; Bunnell et al. 2016). This led to updates in the labeling of TDF‐containing products globally, recommending the monitoring for TDF adverse reactions when LDV/SOF is coadministered or using alternative HCV or HIV therapy. An alternative to TDF is needed for HIV/HCV‐coinfected patients given the known interactions and the potential for additional concerns.

When R/F/TDF is coadministered with LDV/SOF, TFV exposure was 4,780 ng·h/mL representing a 40% increase compared to R/F/TDF alone (German et al. 2014). There was a 75% increase in TFV exposure observed when R/F/TAF is coadministered with LDV/SOF (467 ng·h/mL vs. 268 ng·h/mL). Despite the 75% increase, the absolute TFV exposure value is 10‐fold lower for R/F/TAF compared to R/F/TDF coadministered with LDV/SOF (467 ng·h/mL vs. 4,780 ng·h/mL); this supports that there is no clinically relevant interaction with the coadministration of R/F/TAF and LDV/SOF. This 10‐fold difference is explained by the markedly lower (~90%) TFV exposures expected with TAF 25 mg compared to TDF 300 mg, given the lower dose of the TAF prodrug.

The TFV PK data for R/F/TAF presented here and similar supportive data for the FDC E/C/F/TAF provide HIV health care providers with the option of switching TDF for TAF as a way to address the TDF drug interaction concern with LDV/SOF (Custodio et al. 2015). These data have been incorporated into the DHHS HIV treatment guidelines stating there is the need to monitor for TDF toxicities when LDV/SOF is coadministered with TDF but not with TAF, as well as the AASLD‐IDSA HCV guidelines stating TAF may be an alternative to TDF during LDV/SOF treatment for patients who take cobicistat or ritonavir as part of their ART because LDV increases TFV levels when given as TDF (American Association for the Study of Liver Diseases, 2017; DHHS, 2016). It is expected that TAF may replace TDF in HIV management given the July 2016 IAS‐USA guidelines recommend TAF over TDF; furthermore, the DHHS guidelines include TAF in their recommended regimens based on safety differences stemming from the PK differences between TAF and TDF as well as virologic efficacy.

HIV/HCV‐coinfected patients on R/F/TAF can initiate LDV/SOF without concerns regarding potential drug interaction given the findings of this PK study. TAF has expanded the treatment options available to HIV/HCV‐coinfected patients and provides a safer option compared to TDF. Using a TAF‐based regimen, such as R/F/TAF, instead of a TDF‐based regimen in HIV/HCV‐coinfected patients will avoid the need for additional monitoring or modifying the ARV regimen to accommodate the HCV treatment.

## Disclosure

None declared.
